# Prevalence of non-*falciparum* malaria infections among asymptomatic individuals in four regions of Mainland Tanzania

**DOI:** 10.1186/s13071-024-06242-4

**Published:** 2024-03-23

**Authors:** Zachary R. Popkin-Hall, Misago D. Seth, Rashid A. Madebe, Rule Budodo, Catherine Bakari, Filbert Francis, Dativa Pereus, David J. Giesbrecht, Celine I. Mandara, Daniel Mbwambo, Sijenunu Aaron, Abdallah Lusasi, Samwel Lazaro, Jeffrey A. Bailey, Jonathan J. Juliano, Julie R. Gutman, Deus S. Ishengoma

**Affiliations:** 1 Institute for Global Health and Infectious Diseases, University of North Carolina, Chapel Hill, NC USA; 2 National Institute for Medical Research, Dar es Salaam, Tanzania; 3 National Institute for Medical Research, Tanga Center, Tanga, Tanzania; 4 The Connecticut Agricultural Experiment Station, New Haven, CT USA; 5 National Malaria Control Programme, Dodoma, Tanzania; 6 Department of Pathology and Laboratory Medicine, Warren Alpert Medical School, Brown University, Providence, RI USA; 7 Center for Computational Molecular Biology, Brown University, Providence, RI USA; 8 Malaria Branch, National Center for Emerging and Zoonotic Infectious Diseases, US Centers for Disease Control and Prevention (CDC), Atlanta, GA USA; 9 Harvard T.H. Chan School of Public Health, Boston, MA USA; 10 Faculty of Pharmaceutical Sciences, Monash University, Melbourne, VIC Australia

**Keywords:** Malaria, *Plasmodium malariae*, *Plasmodium ovale*, *Plasmodium vivax*, Non-falciparum species, Tanzania, Asymptomatic malaria

## Abstract

**Background:**

Recent studies point to the need to incorporate the detection of non-falciparum species into malaria surveillance activities in sub-Saharan Africa, where 95% of the world’s malaria cases occur. Although malaria caused by infection with *Plasmodium falciparum* is typically more severe than malaria caused by the non-falciparum* Plasmodium* species *P. malariae*, *P. ovale* spp. and *P. vivax*, the latter may be more challenging to diagnose, treat, control and ultimately eliminate. The prevalence of non-falciparum species throughout sub-Saharan Africa is poorly defined. Tanzania has geographical heterogeneity in transmission levels but an overall high malaria burden.

**Methods:**

To estimate the prevalence of malaria species in Mainland Tanzania, we randomly selected 1428 samples from 6005 asymptomatic isolates collected in previous cross-sectional community surveys across four regions and analyzed these by quantitative PCR to detect and identify the *Plasmodium* species.

**Results:**

*Plasmodium falciparum* was the most prevalent species in all samples, with *P. malariae* and *P. ovale* spp. detected at a lower prevalence (< 5%) in all four regions; *P. vivax* was not detected in any sample.

**Conclusions:**

The results of this study indicate that malaria elimination efforts in Tanzania will need to account for and enhance surveillance of these non-falciparum species.

**Graphical Abstract:**

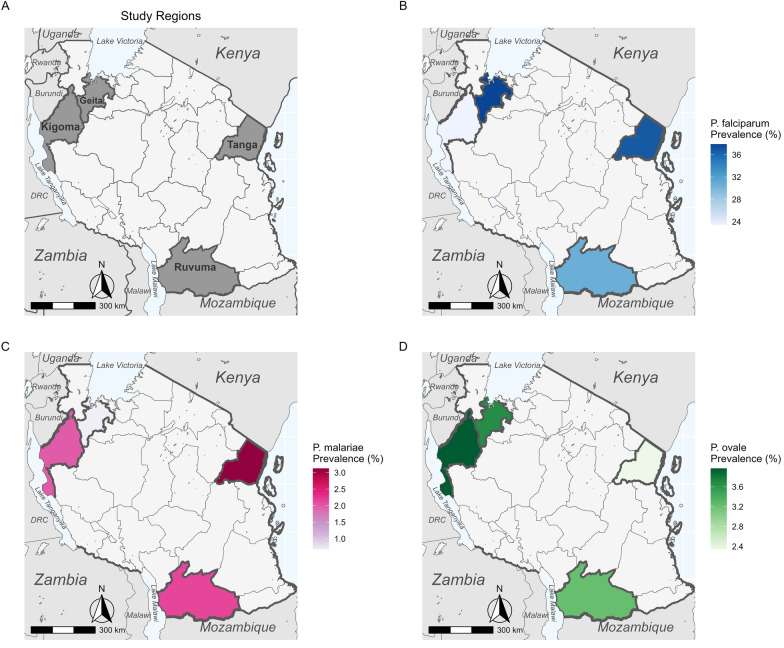

**Supplementary Information:**

The online version contains supplementary material available at 10.1186/s13071-024-06242-4.

Tanzania has one of the highest malaria burdens in the world, accounting for 3.1% of global malaria cases and 4.1% of global malaria deaths in 2021 [1]. While most malaria cases in Tanzania and elsewhere in sub-Saharan Africa are caused by *Plasmodium falciparum*, four other *Plasmodium* species (*P. vivax*, *P. malariae*, *P. ovale curtisi* and *P. ovale wallikeri*) are present to varying degrees. Current data also suggest that the prevalence of these species is higher than previously believed and that they may become even more prevalent as *P. falciparum* is controlled and ultimately eliminated [2–6], in line with the WHO goal of a 90% reduction in global malaria burden from 2015 levels by 2030 [7]. Non-falciparum malaria (i.e. malaria infection due to* Plasmodium* species other than* P. falciparum*) may require different control measures due to major differences in biology, including different *Anopheles* vectors with different seasonal peaks [8], relapse and/or chronic infections [9, 10], lower parasitemia [11] and higher rates of asymptomatic infection [8].

Previous work in Mainland Tanzania has characterized the prevalence of non-falciparum malaria in school children (age: 5–16 years) [6] and non-falciparum positivity rates among symptomatic patients [12]. In the school children, the prevalence of *P. ovale* spp. malaria infections (24%) was similar to that of *P. falciparum* (22%) [6], while in symptomatic patients, *P. falciparum* malaria infections were much more abundant than non-falciparum malaria, although *P. ovale* spp. positivity rates surpassed 5% in seven of 10 regions [12]. In both studies, *P. malariae* was less common than either *P. falciparum* or *P. ovale* spp., and *P. vivax* was rare [6, 12]. However, a full characterization of non-falciparum prevalence in Mainland Tanzania requires accounting for asymptomatic individuals of all ages, who may act as potential reservoirs. In the study reported here, we characterized the prevalence of all malaria species among asymptomatic individuals across all ages in three regions with moderate and high malaria transmission intensity, and in children aged < 5 years in one region with high transmission.

The study protocol was approved by the Tanzanian Medical Research Coordinating Committee (MRCC) of the National Institute for Medical Research (NIMR) and involved approved standard procedures for informed consent and sample deidentification. Additional details are described elsewhere [13]. Deidentified samples were considered non-human subjects’ research at the University of North Carolina and Brown University.

A random subset of 1428 dried blood spot (DBS) samples were drawn from a total of 5860 asymptomatic samples, including 694 samples from a total of 2647 collected from all age groups [12] during cross-sectional community surveys in the Kigoma (*n* = 252/878, high transmission area), Ruvuma (*n* = 186/741, high transmission area) and Tanga (*n* = 256/1028, moderate transmission area) regions during the Molecular Surveillance of Malaria in Tanzania (MSMT) project in 2021 [13]. The random subset was representative of the regional sample distribution (*χ*^2^ = 2.43, *df* = 2, *P* = 0.3; Additional file 1: Table S1), but not representative of the age group distribution (*χ*^2^ = 10.46, *df* = 2, *P* = 0.005; Additional file 1: Table S2), or the sex distribution (*χ*^2^ = 43.65, *df* = 1, *P* < 0.001; Additional file 1: Table S3). An additional 734 samples were drawn from 3213 samples collected from children aged < 5 years during cross-sectional household surveys for the Group Antenatal Care project (GANC) [14, 15] in Geita in 2021. In all regions, study participants were administered a malaria rapid diagnostic test (mRDT) in their communities. Participants with a positive test result were treated with artemether-lumefantrine and adjunct medications based on the presence of concurrent illnesses.

The molecular analyses used to detect *Plasmodium* spp. in each sample are described in detail elsewhere [12]. Briefly, we performed a separate quantitative PCR (qPCR) assay targeting the 18S ribosomal RNA gene (*18S* rRNA) for each species, which allows for both the detection of each species as well as a semi-quantitative estimate of parasitemia. For each region, we calculated prevalence for each species, including both single-species and mixed-species infections. Regional-level maps of prevalence for each species were created using the R package *sf* (version 1.0.9; R Foundation for Statistical Computing, Vienna, Austria) based on shape files available from GADM.org and naturalearthdata.com accessed via the R package *rnaturalearth* (version 0.3.2) [16]. Variation in species-specific prevalence by region and age group was assessed for significance with generalized linear models (GLMs) or analysis of variance (ANOVA), as appropriate, in R.

Excluding the Geita participants who were all aged < 5 years and whose ages were not recorded, the median age of the remaining 694 participants was 20 (interquartile range [IQR] 8–47) years (range 6 months to 87 years). Including the Geita participants, children (≤ 16 years old) constituted 74.2% of participants (*n* = 1060), while adults (> 16 years old) constituted the remaining 25.8% (*n* = 368). Young children (< 5 years old) comprised 77.9% (*n* = 826) of the pediatric participants, while the remaining 22.1% (*n* = 234) were school-aged children (5–16 years old). Sex identifications were available for 694 participants and were female-skewed, with 505 female (72.8%) and 189 male participants (27.2%). Of the sampled individuals, 21.1% (*n* = 301) were RDT-positive (Additional file 1: Table S4) using a standard HRP2/Pan RDT (First Response® [Premier Medical Corp., Ltd, Valsad, Gujarat, India]; SD Bioline™ [Abbott Diagnostics Ltd. Korea, Seoul, Republic of Korea]; or Care Start™ [Access Bio, Inc., Somerset, NJ, USA]).

Among all 1428 samples analyzed, *P. falciparum* was detected in 34.2% (*n* = 488, 95% confidence interval [CI] 31.7–36.7%), *P. malariae* in 1.5% (*n* = 22, 95% CI 0.99–2.4%) and *P. ovale* spp. in 3.4% (*n* = 49, 95% CI 2.6–4.5%); *P. vivax* was not detected in any sample. *Plasmodium malariae* infections were nearly evenly split between single-species infections (45.5%, *n* = 10/22) and mixed-species infections with *P. falciparum* (40.9%, *n* = 9/22), with the remaining three infections (13.6%) being triple infections with *P. falciparum* and *P. ovale* spp. (Table 1). In contrast, most *P. ovale* spp. infections were mixed with *P. falciparum* (65.3%, *n* = 32/49), with single-species infections being less common (28.6%, *n* = 14/49) and triple infections comprising the remainder (Table 1). The highest median parasitemia was recorded for *Plasmodium malariae* at 164,080 parasites/µl blood (IQR 9942–1333,100 p/µl), followed by *P. falciparum* at 55,200 (IQR 2910–775,000) and *P. ovale* spp. at 11,868 (IQR 1271–70,840) p/µl. However, there was no significant difference in parasitemia by species (*F*_(2,556)_ = 0.085, *P* = 0.9).

**Table 1:** Infection composition proportions for isolates infected with *Plasmodium malariae* and *Plasmodium ovale* spp.

Infection type^a^	Count (*n*)	Proportion (%)
All* Pm* infections	22	
*Pm*	10	45.5
*Pm/Pf*	9	40.9
*Pm/Pf/Po*	3	13.6
All *Po* infections	49	
*Po*	14	28.6
*Po/Pf*	32	65.3
*Pm/Pf/Po*	3	6.1

Sum of proportions may not equal 100% due to rounding

*Pf Plasmodium falciparum*,* Pm* *P. malariae*,* Po* *P. ovale* spp.

^a^Refers to single-species infections and mixed-species infections (2 or 3* Plasmodium *spp.)

All three* Plasmodium* species recorded in the samples (*P. vivax* was not detected in any sample) were detected in each region (Fig. 1). The highest *P. falciparum* prevalence was found in Geita and Tanga (37.8% [*n* = 278/734] and 36.7%, [94/256], respectively; Additional file 1: Table S5). *Plasmodium malariae* was relatively rare in all four regions, with the highest prevalence recorded in Tanga (3.1%, *n* = 8/256) and the lowest in Geita (0.7%, *n* = 5/734; Additional file 1: Table S5). *Plasmodium ovale* spp. prevalence was slightly higher than that of *P. malariae* (3.2–4.0%, *n* = 6/186–10/252) in all regions except Tanga (2.3%, *n* = 6/256; Additional file 1: Table S5). There was significant variation (by ANOVA) in prevalence between regions for *P. falciparum* (*F*_(3,1424)_ = 6.47,* P* < 0.001) and *P. malariae* (*F*_(3,1424)_ = 2.87,* P* = 0.0351), but not for *P. ovale* spp. (*F*_(3,1424)_ = 0.43,* P* = 0.732).

**Fig. 1:**
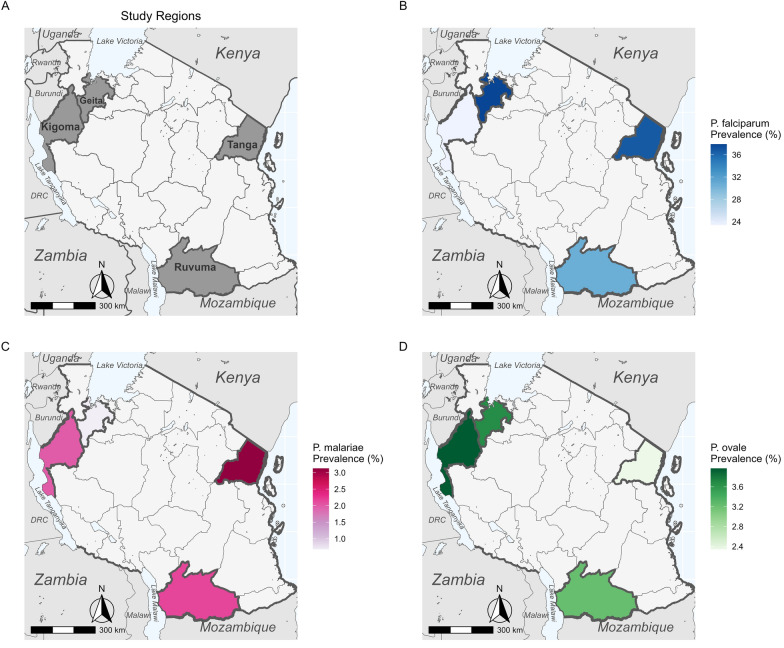
Maps of Tanzania showing the location of study regions (**a**) and the regional prevalence of *Plasmodium falciparum* (**b**), *P. malariae* (**c**) and *P. ovale* spp. (**d**). *Plasmodium vivax* was not detected in any sample and therefore was not mapped

While age was a significant (GLM: *t*_(1427)_ = 23.7_,_ *P* < 0.001) determinant of *P. falciparum* infection, there was no significant effect of age for infection with either *P. malariae* or *P. ovale* spp. Age group was found to be a significant determinant of infection likelihood for both *P. falciparum* (*F*_(2,1418)_ = 4.92, *P* = 0.007) and *P. malariae* (*F*_(3,1418)_ = 3.28,* P* = 0.0381), but not for *P. ovale* spp. (*F*_(3,1418)_ = 0.857,* P* = 0.425) (Fig. 2). While children were significantly (Tukey HSD test: *P* < 0.05) more likely than adults to have *P. falciparum* infection, adults were more likely to have *P. malariae* infection (Tukey HSD test: *P* < 0.05; Fig. 2). There was no significant interaction between age group and region for either *P. falciparum* or *P. malariae* infection, but the interaction was nearly significant (*P* = 0.055) for *P. ovale* spp. infection

**Fig. 2:**
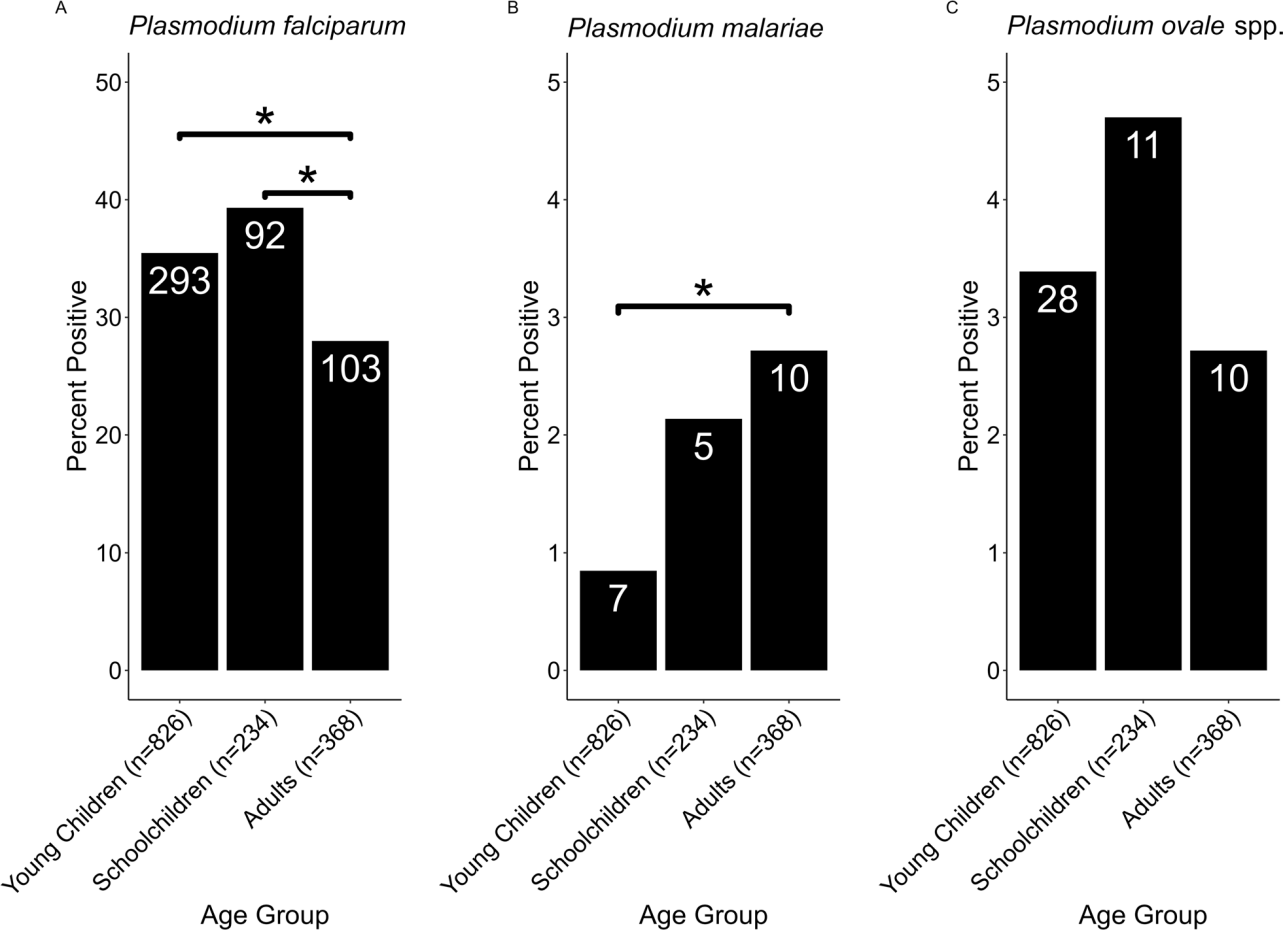
Tukey HSD analysis of malaria species prevalence by age group. A total of 826, 234 and 368 samples were positive for malaria infection in the age groups ‘young children’ (< 5 years), ‘school children’ (5–16 years) and ‘adults’ (> 16 years), respectively. The total number of samples per group for each species is shown on the* X*-axis under each bar, and the number of positive samples for each group is shown in each bar.** a**,** b**,** c** Prevalence by age group of *P. falciparum* (**a**) *P. malariae* (**b**) and *P. ovale* spp. (**c**). All comparisons marked with an asterisk were significant at the *P* < 0.05 level; all other comparisons were statistically non-significant

This study builds on previous research with school children and patients with a clinical diagnosis of malaria to describe the prevalence of different malaria species within four regions of Mainland Tanzania. Although *P. falciparum* is the most prevalent species in Mainland Tanzania, we found that the prevalence of both *P. malariae* and *P. ovale* spp. surpassed 3% in at least one region, and could increase as *P. falciparum* is locally eliminated, as has been seen with non-falciparum species in other contexts [1, 4]. In contrast to a 2017 study involving school children, which found that the prevalence of *P. ovale* spp. was similar to that of *P. falciparum* [6], we found *P. ovale* spp. prevalence to be much lower than that of *P. falciparum*. In addition, the 2017 survey of school children [6] mostly identified *P. ovale* spp. as single-species infections and *P. malariae* as mixed infections with *P. falciparum*. In contrast, most of our samples with *P. ovale* spp. infection were mixed with *P. falciparum*, and we found similar proportions of single-species and mixed-species *P. malariae* infections. However, our sample sizes are small and may not necessarily be representative of the full picture, particularly in Geita where samples were only collected from children aged < 5 years. Also, our study included only four regions, of which only one (Tanga) overlaps with those included in the previous study, although we did include a wider age range.

In Tanga, we may have found lower *P. ovale* spp. prevalence than that reported in the previous study due to the inclusion of adults in our study, who are less likely to test positive for this species, whereas schoolchildren are a major asymptomatic infectious reservoir [17–19]; however, we did not replicate a significant difference in this study. In addition, the disruptions to malaria control caused by the COVID-19 pandemic, particularly in 2020–2021 [20], could have caused an increase in *P. falciparum* prevalence, which might account for the decreased prevalence of *P. ovale* if there is competition between the two species. Indeed, there was no difference in malaria prevalence in the villages of Magoda, Mamboleo and Mpapayu between 2019 (24.9%) and 2021 (24.5%; unpublished data). However, the prevalence dropped to 6.4% in 2022 following a return to the implementation of normal control activities (unpublished data), so further longitudinal studies may clarify the impact of resumed intense *P. falciparum* control. However, the malaria prevalence in Tanga dropped from 34.8% to 26.2% between 2020 and 2021 (unpublished data), so *P. falciparum* control in this region was likely effective during the course of this study, meaning that other factors, such as the inclusion of adults or rainfall levels, were likely a larger determinant of the lower *P. ovale* spp. prevalence in our study.

We did not find school children to be significantly more likely than adults or young children to test positive for either *P. malariae* or *P. ovale* spp. [12], but this trend, although not significant, was recorded for *P. ovale* spp. prevalence in this study. Therefore, the lack of significance in these species is likely an artifact of small sample sizes (Table 1; Fig. 2, Additional file 2: Fig. S1). Our finding that *P. malariae* was significantly more prevalent among adults likely reflects the presence of chronic infections that are more likely to be found in adults than in children due to the inherently larger number of infection opportunities [10].

Although *P. falciparum* remains the most prevalent species in these four regions, *P. malariae* and *P. ovale* spp. are present in all four regions, whereas *P. vivax* was not detected. Achieving malaria elimination in Tanzania will require ongoing surveillance of and targeted interventions for these species. While standard treatments successfully clear *P. falciparum*, the 3.4% of patients in this study with *P. ovale* spp. malaria infection may relapse. This study serves as a complement to previous studies focusing on school children and symptomatic patients and provides a full picture of the non-falciparum malaria landscape for communities in Mainland Tanzania. Ongoing analysis of samples collected in 2022 and 2023 will allow us to detect temporal trends in prevalence, and a forthcoming genomic analysis of *P. malariae* and *P. ovale* spp. isolates from Tanzania will inform our understanding of population structure and diversity in these species.

### Supplementary Information

**Additional file 1: Table S1.** Regional distribution of samples in random subset and full dataset. **Table S2.** Age group distribution of samples in random subset and full dataset. **Table S3.** Sex distribution of samples in random subset and full dataset. **Table S4.** Species positivity by region and age group.

**Additional file 2: Figure S1.** Samples included in analysis by age group and region.

## Data Availability

Data are available upon reasonable request to the corresponding author.
