# Supplementary material for: Prevalence of non-falciparum malaria infections among asymptomatic individuals in four regions of Mainland Tanzania
Source: Parasit Vectors. 2024 Mar 23;17:153. doi: 10.1186/s13071-024-06242-4 (PMC10960463; doi:10.1186/s13071-024-06242-4)
Supplement: Supplementary file 1 — Additional file 1: Table S1. Regional distribution of samples in random subset and full dataset. Table S2. Age group distribution of samples in random subset and full dataset. Table S3. Sex distribution of samples in random subset and full dataset. Table S4. Species positivity by region and age group. [file 13071_2024_6242_MOESM1_ESM.docx]

Table S1 – Regional Distribution of Samples in Random Subset and Full Dataset

| **Region** | **Subset** | **Full Dataset** |
| --- | --- | --- |
| Kigoma | 252 (36.3%) | 878 (33.2%) |
| Ruvuma | 186 (26.8%) | 741 (28.0%) |
| Tanga | 256 (36.9%) | 1,028 (38.8%) |
| **Total Samples** | **694** | **2,647** |

Table S2 – Age Group Distribution of Samples in Random Subset and Full Dataset

| **Age Group** | **Subset** | **Full Dataset** |
| --- | --- | --- |
| Young Children (<5 years) | 92 (13.3%) | 450 (17.0%) |
| Schoolchildren (5-16 years) | 234 (33.7%) | 962 (36.3%) |
| Adults (>16 years) | 368 (53.0%) | 1,235 (46.7%) |
| **Total Samples** | **694** | **2,647** |

Table S3 – Sex Distribution of Samples in Random Subset and Full Dataset

| **Sex** | **Subset** | **Full Dataset** |
| --- | --- | --- |
| Female | 505 (72.8%) | 1,568 (59.3%) |
| Male | 189 (27.2%) | 1,078 (40.7%) |
| **Total Samples** | **694** | **2,646*** |

*Sex was not reported for all individuals.

Table S4 – Comparison of qPR and RDT Results by Diagnosis

| **Diagnosis** | **RDT Negative** | **RDT Positive** |
| --- | --- | --- |
| Pf | 233 (52.5%) | 211 (47.5%) |
| Pm | 10 (100%) | 0 |
| Pm/Pf | 3 (33.3%) | 6 (66.7%) |
| Pm/Pf/Po | 1 (33.3%) | 2 (66.7%) |
| Po | 14 (100%) | 0 |
| Po/Pf | 6 (18.8%) | 26 (81.3%) |
| Negative | 857 (93.6%) | 59 (6.4%) |

Table S5 **–** Species Positivity by Region and Age Group

| **Region** | **Age Group** | **Pf-Positive Samples** | **Pm-Positive Samples** | **Po-Positive Samples** | **Samples Tested** |
| --- | --- | --- | --- | --- | --- |
| Geita* | Young Children | 278 (37.9%) | 5 (0.7%) | 27 (3.7%) | 734 |
|  | Schoolchildren | * | * | * | * |
|  | Adults | * | * | * | * |
|  | **All Ages** | **278 (37.9%)** | **5 (0.7%)** | **27 (3.7%)** | **734** |
| Kigoma | Young Children | 7 (18.4%) | 1 (2.6%) | 1 (2.6%) | 38 |
|  | Schoolchildren | 26 (34.2%) | 2 (2.6%) | 7 (9.2%) | 76 |
|  | Adults | 26 (18.8%) | 2 (1.4%) | 2 (1.4%) | 138 |
|  | **All Ages** | 59 (23.4%) | 5 (2.0%) | 10 (4.0%) | 252 |
| Ruvuma | Young Children | 0 | 0 | 0 | 23 |
|  | Schoolchildren | 22 (37.9%) | 2 (3.4%) | 3 (5.1%) | 58 |
|  | Adults | 35 (33.3%) | 2 (1.9%) | 3 (2.9%) | 105 |
|  | **All Ages** | 57 (30.6%) | 4 (2.2%) | 6 (3.2%) | 186 |
| Tanga | Young Children | 8 (25.8%) | 1 (3.2%) | 0 | 31 |
|  | Schoolchildren | 44 (44%) | 1 (1%) | 1 (1%) | 100 |
|  | Adults | 42 (33.6%) | 6 (4.8%) | 5 (4%) | 125 |
|  | **All Ages** | 94 (36.7%) | 8 (3.1%) | 6 (2.3%) | 256 |

*****Only children under five were enrolled in the study in Geita. Schoolchildren are defined as ages 5-16, and adults are all those >16 years.
