# Supplementary figures and images for: Prevalence of non-falciparum malaria infections among asymptomatic individuals in four regions of Mainland Tanzania
Source: Parasit Vectors. 2024 Mar 23;17:153. doi: 10.1186/s13071-024-06242-4 (PMC10960463; doi:10.1186/s13071-024-06242-4)

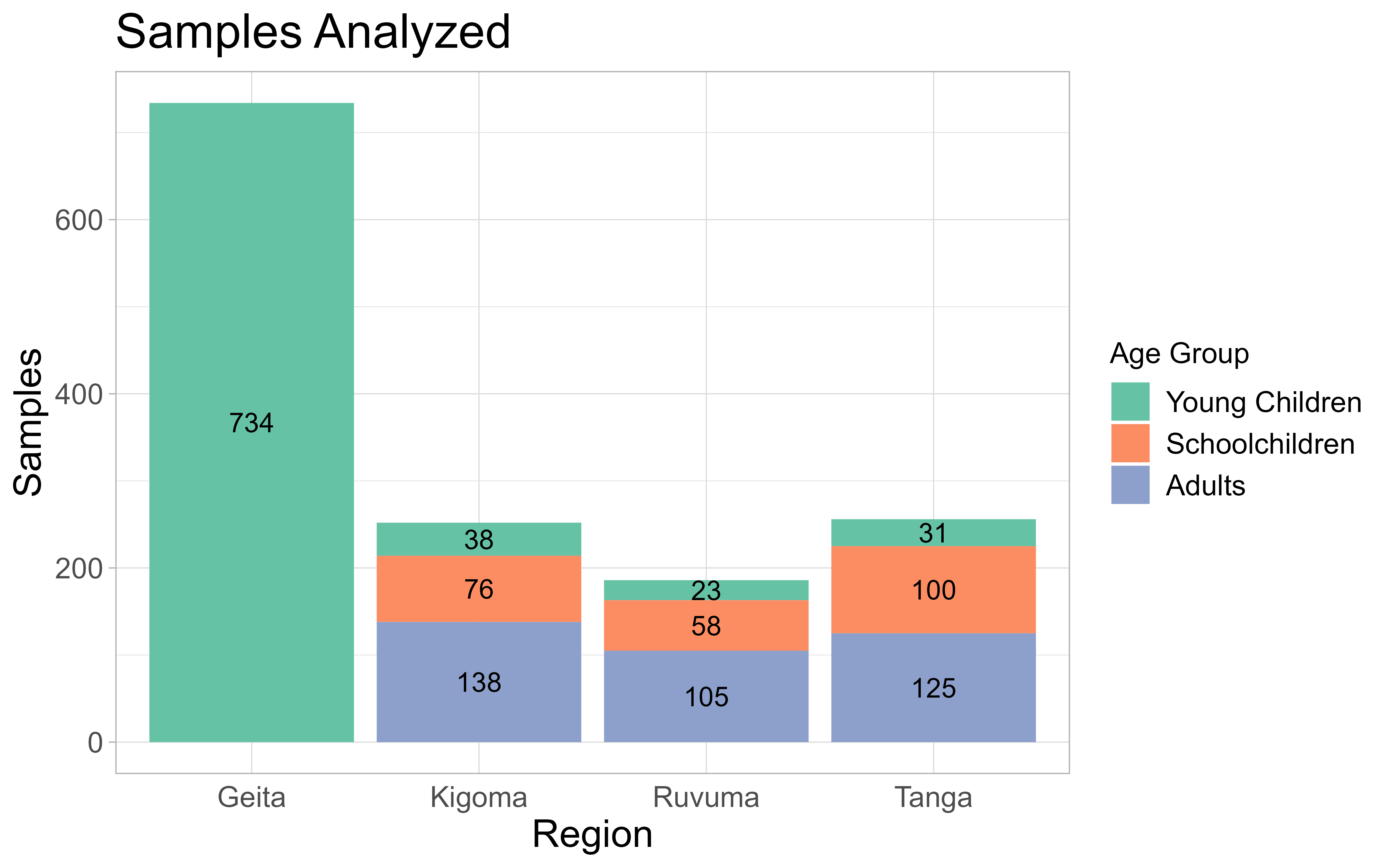

Supplement: Supplementary file 2 — Additional file 2: Figure S1. Samples included in analysis by age group and region. [file 13071_2024_6242_MOESM2_ESM.png]
